# Induction of Highly Functional Hepatocytes from Human Umbilical Cord Mesenchymal Stem Cells by HNF4α Transduction

**DOI:** 10.1371/journal.pone.0104133

**Published:** 2014-08-19

**Authors:** Hualian Hang, Yabin Yu, Ning Wu, Qingfeng Huang, Qiang Xia, Jianmin Bian

**Affiliations:** 1 Department of Liver Surgery, Ren Ji Hospital, School of Medicine, Jiao Tong University, Shanghai, China; 2 Department of General Surgery, Nanjing Hospital Affiliated to NanJing Medical University, Nanjing, China; Wake Forest Institute for Regenerative Medicine, United States of America

## Abstract

**Aim:**

To investigate the differentiation potential of human umbilical mesenchymal stem cells (HuMSCs) and the key factors that facilitate hepatic differentiation.

**Methods:**

HuMSCs were induced to become hepatocyte-like cells according to a previously published protocol. The differentiation status of the hepatocyte-like cells was examined by observing the morphological changes under an inverted microscope and by immunofluorescence analysis. Hepatocyte nuclear factor 4 alpha (HNF4α) overexpression was achieved by plasmid transfection of the hepatocyte-like cells. The expression of proteins and genes of interest was then examined by Western blotting and reverse transcription-polymerase chain reaction (RT-PCR) or real-time RT-PCR methods.

**Results:**

Our results demonstrated that HuMSCs can easily be induced into hepatocyte-like cells using a published differentiation protocol. The overexpression of HNF4α in the induced HuMSCs significantly enhanced the expression levels of hepatic-specific proteins and genes. HNF4α overexpression may be associated with liver-enriched transcription factor networks and the Wnt/β-Catenin pathway.

**Conclusion:**

The overexpression of HNF4α improves the hepatic differentiation of HuMSCs and is a simple way to improve cellular sources for clinical applications.

## Introduction

Most liver diseases lead to hepatocyte dysfunction with possible eventual organ failure. Bioartificial livers and hepatocyte transplantation have been regarded as useful bridges for patients waiting for whole-organ transplantation [1], [2] by providing metabolic support during liver failure. However, organ shortage remains a major limiting step of this procedure. More recent developments in stem cell technology have highlighted a new source of liver cells for use in regenerative medicine. In particular, mesenchymal stem cells (MSCs), due to their multipotency and low immunogenicity, have been suggested as an ideal population of stem cells for the treatment of liver injury. Human umbilical cord mesenchymal stem cells (HuMSCs) isolated from umbilical cord Wharton's jelly are more primitive MSCs than those isolated from other tissue sources and do not express the major histocompatibility complex (MHC) class II (HLA-DR) antigens [3], [4]. However, the hepatic differentiation status of hepatocyte-like cells derived from stem cells is not sufficient for clinical use because of the relatively low expression levels of functional proteins and the lack of full induction of metabolic activity [5]. Therefore, it is important to define a method to promote hepatic maturation.

Hepatocyte differentiation is associated with changes in gene expression that are primarily controlled at the level of transcription. Studies of the transcriptional gene regulatory elements expressed in hepatocytes have identified a number of liver transcription factors, including hepatocyte nuclear factor (HNF)-1, -3, -4, and -6 and the CCAAT/enhancer-binding protein (CEBP) family, that are capable of modulating hepatocyte gene expression in hepatoma cells [6], [7]. Hepatocyte nuclear factor 4 alpha (HNF4α), an important transcription factor of the nuclear hormone receptor family, is essential for normal liver architecture, the morphological and functional differentiation of hepatocytes, and the generation of the hepatic epithelium [8], [9]. Several studies have demonstrated that HNF4α may act as a master gene in a transcription factor cascade that could drive hepatic differentiation [10], [11]. The overexpression of HNF4α could improve the hepatocyte functions of hepatocyte-like cells derived from human embryonic stem cells, induced pluripotent stem cells and bone marrow mesenchymal stem cells [12], [13]. In this study, we demonstrate that HuMSCs can be differentiated into hepatocyte-like cells according to the protocol established by Lee et al. [14]. The overexpression of HNF4α can significantly improve the differentiation status of hepatocyte-like cells through the activation of several target genes. These more differentiated hepatocyte-like cells can provide a better cell source for future clinical applications.

## Materials and Methods

### Isolation and culture of HuMSCs

With the informed consent of the tissue donor, and following the ethical and institutional guidelines, fresh human umbilical cords were obtained from male or female fetuses after birth, and 20 cords were collected in our experiment. The study was approved by the Institution Review Board and Human Ethics Committee of Affiliated Nanjing Hospital of Nanjing Medical University, Jiangsu, China. Written consent for the use of the samples for research purposes was obtained from all of the included patients. The samples were then maintained in phosphate-buffered saline (PBS; Invitrogen, Carlsbad, CA, USA) containing 100 U/mL penicillin(Sigma-Aldrich, St Louis, MO, USA) and 100 mg/mL streptomycin(Sigma-Aldrich) at 4°C. Following disinfection in 75% ethanol for 1 min, the umbilical cord vessels were removed in PBS. HuMSCs were prepared as previously described [15]. The mesenchymal tissue was diced into cubes of approximately 1 cm^3^. Following the removal of the supernatant fraction, the precipitate was washed with DMEM (Invitrogen) and centrifuged at 250×g for 5 min. The mesenchymal tissue was treated with collagenase II (Invitrogen) at 37°C for 1 h and further digested with 0.25% trypsin (Invitrogen) at 37°C for 30 min. Fetal bovine serum (FBS; Invitrogen) was added to the mesenchymal tissue to neutralize the excess trypsin. The dissociated mesenchymal cells were further dispersed by treatment with 10% FBS-DMEM and counted. The mesenchymal cells were then used directly for cultures, and the medium was changed twice a week.

### Flow cytometry analysis

Antibodies against the human antigens CD13, CD105, CD90, CD34, CD45, HLA-DR and HLA-DQ were purchased from BD Sciences (Shanghai, CHINA). A total of 1×10^6^ cells were resuspended in 200 µL of PBS and incubated with FITC- or PE-conjugated antibodies for 30 min at room temperature. The fluorescence intensity of the cells was evaluated by flow cytometry using a flow cytometer (FACScan; BD Sciences), and the data were analyzed using CELLQUEST Pro software (BD Sciences).

### Adipogenic differentiation

To induce adipogenic differentiation, the cells were treated with adipogenic medium for three weeks with medium changes twice weekly. Briefly, after the cells reached 70% confluence, the medium was changed to adipogenic differentiation medium consisting of L-DMEM supplemented with 10% FBS, 2 mM IBMX (Sigma-Aldrich) and 5 µg/mL insulin solution (Sigma-Aldrich). The generation of lipid vacuoles was visualized by staining with Oil Red O (Sigma-Aldrich).

### Pancreatic differentiation

The pancreatic induction of HuMSCs was performed according to the procedure developed by Wang et al [16]. The cells were cultured for seven days in DMEM/F12 (Invitrogen) medium containing 10% FBS, 4 nM activin-A (Sigma-Aldrich), 10 mM nicotinamide (Sigma-Aldrich) and 25 ng/mL epidermal growth factor (EGF, PeproTech, Rocky Hill, NJ, USA). The culture medium was changed to DMEM/F12 for seven days. Finally, 10 mM nicotinamide, insulin/transferring/selenium (ITS, Invitrogen), and 10 ng/mL basic fibroblastic growth factor (bFGF, PeproTech) were added, and the incubation was continued for 17 days. After induction, the cells were stained with dithizone.

### Chondrogenic Differentiation

To induce Chondrogenic differentiation, the 4th passage cells were treated with Chondrogenic medium for 21 days (A1007101 STEMPRO CHONDRO DIFF KIT, GIBCO). Medium changes were performed twice weekly, and chondrogenesis was assessed by immunohistochemical staining for type II collagen.

### Hepatic differentiation of HuMSCs

According to a previously described protocol [14], the stem cells were deprived for two days in IMDM supplemented with 20 ng/mL EGF, 10 ng/mL bFGF and 0.61 g/mL nicotinamide for seven days. The cells were then treated with step-2 maturation medium consisting of IMDM supplemented with 20 ng/mL oncostatin M (OSM, PeproTech), 1 µmol/L dexamethasone (DEX, Sigma-Aldrich), and 50 mg/mL ITS. The medium was changed twice per week.

### Immunofluorescence analysis

The cells were fixed in PBS containing 4% paraformaldehyde (NJ-reagent, NanJing, CHINA) for 30 min and permeabilized with phosphate-buffered saline containing 0.1% Triton X-100 (NJ-reagent) for 20 min. The samples were incubated with anti-HNF4α antibody (1∶200; Santa Cruz Biotechnology, Santa Cruz, CA, USA), anti-human serum AFP antibody (1∶200; Santa Cruz Biotechnology), and anti-human serum ALB antibody (1∶200;Santa Cruz Biotechnology), and then with the secondary antibody conjugated to fluorescent phycobiliproteins, namely DyLight 594- and Alexa 488-conjugated goat anti-mouse immunoglobulin G (1∶1000; Beyotime, Shanghai, China). DAPI (Beyotime) was used for nuclear counterstaining.

### Glycogen Storage

Intracellular glycogen was analyzed by Periodic AcidSchiff (PAS) staining. Culture dishes containing the cells were fixed in 4% paraformaldehyde and permeabilized with 0.1% Triton X-100 for 10 min. The samples were then oxidized in 1% periodic acid for 10 min, rinsed 3 times in deionized water (dH2O), treated with Schiff 's reagent for 20 min at room temperature and rinsed in dH2O for 5 to 10 min. The nuclei were stained with Mayer's hematoxylin for 1 min, rinsed in dH2O and assessed under a light microscope.

### Albumin Secretion

The concentration of human Albumin protein on was assayed by a quantitative enzyme-linked immunosorbent assay kit (ELISA) (R&D Systems, USA).

### Expression of MHC

The expression of MHC after differentiation was assayed by Flow cytometry analysis.

### Western blot analysis

The western blot analysis was performed as described previously [17]. The total cellular protein was extracted using cell lysis buffer (KeyGEN, Nanjing, China). The proteins were separated by electrophoresis (Bio-Rad, CA, USA) and transferred to membranes (NJ-reagent). The membranes were blocked in blocking solution (NJ-reagent) and incubated with mouse monoclonal antibodies against alpha-fetoprotein (AFP), albumin (ALB) and cytochrome P450 3A4 (CYP3A4, 1∶200; Santa Cruz Biotechnology) for 1 h at room temperature. After washing, the membranes were incubated for 2 h with horseradish peroxidase (HRP)-linked goat anti-mouse IgG (1∶1000; Biosynthesis, Beijing, China). The membranes were rinsed for 10 s in substrate buffer (NJ-reagent) to remove any residual detergent. A mouse monoclonal antibody against β-actin (1∶5000; KeyGEN) was used as a housekeeping control.

### Reverse transcription-polymerase chain reaction and real-time reverse transcription-polymerase chain reaction analysis

The total RNA from the cells was isolated using the TRIzol reagent (Invitrogen) according to the manufacturer's instructions. The cDNA templates were obtained using oligo(dT) primers and PrimeScript RTase reverse transcriptase (Takara). The products were then subjected to either reverse transcription-polymerase chain reaction (RT-PCR) analysis or quantitative real-time RT-PCR analysis with the specific primer pairs and conditions listed in Table 1.

**Table 1:** Sequences of PCR and real-time PCR primers.

Genes	Sequences of primers	Fragment length (bp)	Annealing temperature (°C)
ALB	F:5′-TGCTTGAATGTGCTGATGACAGGG-3′	162	60
	R: 5′-AAGGCAAGTCAGCAGGCATCTCATC-3′		
AFP	F: 5′-TGCAGCCAAAGTGAAGAGGGAAGA-3′	216	60
	R: 5′-CATAGCGAGCAGCCCAAAGAAGAA-3′		
TAT	F: 5′-TGAGCAGTCTGTCCACTGCCT-3′	359	60
	R: 5′-ATGTGAATGAGGAGGATCTGAG-3′		
G-6P	F: 5′-GCTGGAGTCCTGTCAGGCATTGC-3′	349	60
	R: 5′-TAGAGCTGAGGCGGAATGGGAG-3′		
CK18	F: 5′-GATCGACCTGGACTCCATGAGAA-3′	99	60
	R: 5′-CCGTTGAGCTGCTCCATCTGTA-3′		
CYP3A4	F: 5′-TGTGCCTGAGAACACCAGAG-3′	202	60
	R: 5′-GCAGAGGAGCCAAATCTACC-3′		
α1AT	F: 5′-TCGCTACAGCCTTTGCAATG-3′	142	55
	R: 5′-TTGAGGGTACGGAGGAGTTCC-3′		
Transferrin	F: 5′-ACTAAGTGCCAGAGTTTCCG-3′	130	54
	R: 5′-CAGCATCCGCTTCGTTT-3′		
HNF4α	F: 5′-GCACCAACCTCAACGC-3′	313	56
	R: 5′-AGGCTGCTGTCCTCATAG-3′		
HNF3β	F: 5′-GCACCTGCAGATTCTGATTTT-3′	66	60
	R: 5′-GACTTCCCTGCAACAACAGC-3′		
HNF6	F: 5′-CCTGGAGCAAACTCAAATCC-3′	116	60
	R: 5′-TTCTTTCCTTTTGCATGCTG-3′		
CEBPα	F: 5′-CAACACTTGTATCTGGCCTCTG-3′	112	60
	R: 5′-CGAGCAAAACCAAAACAAAAC-3′		
β-Catenin	F: 5′-ATTGTCCACGCTGGATTTTC-3′	142	58
	R: 5′-AGGTCTGAGGAGCAGCTTCA-3′		
GSK3β	F: 5′-CACCACTGTTGTCACCTTGC-3′	149	60
	R: 5′-AAAGGTGATTCGCGAAGAGA-3′		
DKK4	F: 5′-TAGCACAGAACGGCTTCTCA-3′	143	60
	R: 5′-AGCTCTGGTCCTGGACTTCA-3′		
LEF1	F: 5′-TCACTGTAAGTGATGAGGGGG-3′	150	60
	F: 5′-TCACTGTAAGTGATGAGGGGG-3′		
GAPDH	F:5′-AGGTGAAGGTCGGAGTCAAC-3′	232	52
	F:5′-AGGTGAAGGTCGGAGTCAAC-3′		

### Construction of the PCDNA3.1/HNF4α recombinant plasmid

We generated the HNF4α cDNA using specific primers (forward primer: 5′-AGGATCCATGCGACTCTCCAAAACCCTCG-3′; reverse primer: 5′-AGAATTCCCTAGATAACTTCCTGCTGCTTGGTG-3′). The sequence was then inserted into PCDNA3.1 (Invitrogen), resulting in the generation of PCDNA3.1/HNF4α. The element was confirmed by digesting with EcoR I (TaKaRa, Shiga, Japan) and BamH I (TaKaRa). The two-week-induced MSCs were transfected with the recombinant plasmid using Lipofectamine 2000 (Invitrogen). The target gene overexpression was confirmed by RT-PCR and immunofluorescence.

### Statistical analysis

The statistical significance of the induction effect was determined by t-tests. Differences were considered significant if the P value was less than 0.05.

## Results

### Isolation and culture of HuMSCs

**Figure 1:**
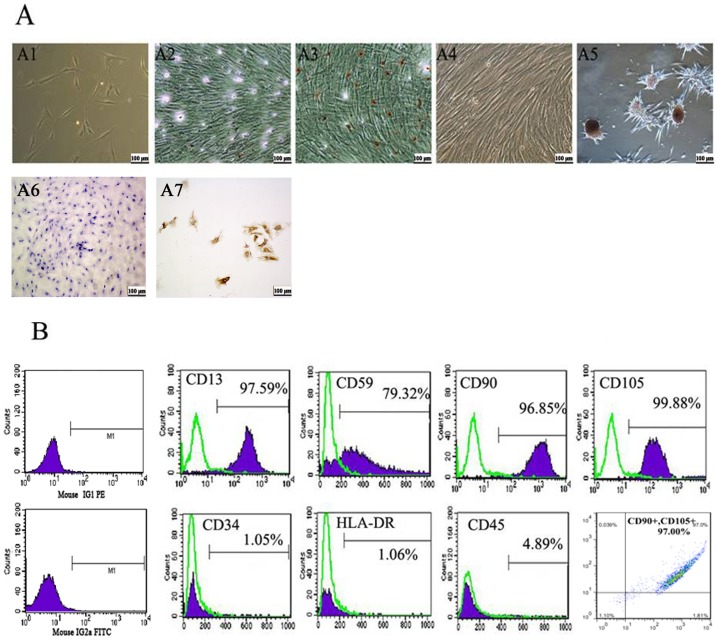
Characterization of HuMSCs. *(* ***A*** *)* A1: Morphology of HuMSCs. Phenotypic characterization of HuMSCs at low confluency; A2: Control; A3: HuMSCs were induced to differentiate into adipogenic cells and stained positive for Oil Red O; A4: Control; A5: Under pancreatic differentiation conditions, HuMSCs formed islet-like structures and stained positive for dithizone. Original magnification, ×100 (A1, A2, A3, A4, A5). A7: Control;A8: Under chondrogenic differentiation conditions, HuMSCs differentiate into chondrogenic cells and immunohistochemical stained positive for type II collagen. *(* ***B*** *)* Characterization of HuMSC marker expression by flow cytometry analysis. HuMSCs were stained with PE- or FITC-conjugated antibodies.

The isolated cord cells manifested heterogeneity during the first five days. Forty-eight hours after plating, the cells were adherent, elongated and spindle-shaped, and the individual colonies formed displayed a fibroblast-like morphology (Fig. 1A1). The primary culture cells reached confluence approximately two weeks later, and the cells were then passaged at a ratio of 1∶3. The cultured HuMSCs were positive for CD13, CD90, CD105 and CD59 but negative for hematopoietic makers, including CD34, CD45 and HLA-DR (Fig. 1B). The HuMSCs were investigated to determine their potential for mesodermal and endodermal differentiation. Oil Red O staining revealed that the HuMSCs contained positively stained lipid droplets in the cytoplasm after adipogenic differentiation (Fig. 1A3). Positive dithizone staining indicated that the HuMSCs could differentiate into pancreatic islet-like cells (Fig. 1A5). Immunohistochemical staining for type II collagen revealed that the HuMSCs could differentiate into cartilage cells after chondrogenic differentiation (Fig. 1A7). Undifferentiated HuMSCs cultured in the growth medium did not show staining for Oil Red O, dithizone or type II collagen. (Fig. 1A2, A4, A6).

### Differentiation status of hepatocyte-like cells induced from HuMSCs

**Figure 2:**
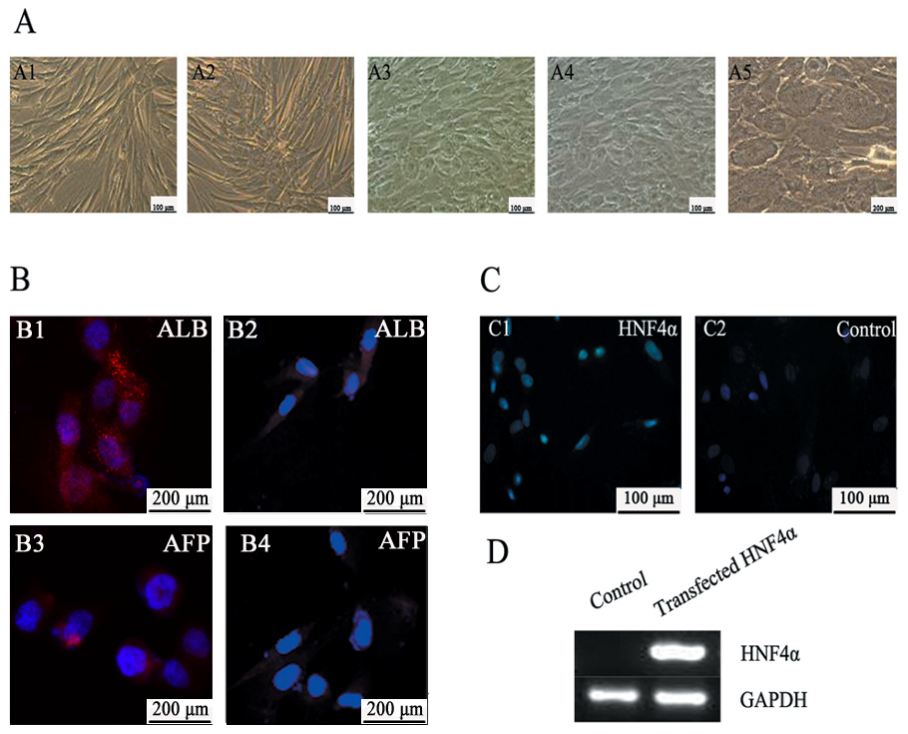
In vitro hepatic differentiation of HuMSCs and HNF4α transfection. *(* ***A*** *)* Morphological changes observed as undifferentiated HuMSCs differentiated into hepatocytes under hepatogenic conditions. A1: Undifferentiated HuMSCs; A2: 1 week postinduction. A3: 2 weeks postinduction. A4–A5: 3 weeks postinduction. Original magnification, ×100 (A1–A4), ×200 (A5). *(* ***B*** *)* Immunocytochemical analysis of hepatocyte-specific marker expression. HuMSCs were cultured in hepatocyte differentiation medium for 21 days (B1, B3) or in growth medium (negative controls; B2, B4). The cells were stained with mouse antibodies against the hepatocyte-specific markers ALB (B1, B2) and AFP (B3, B4) and incubated with Dylight594-conjugated anti-mouse IgG. The cell nuclei were counterstained with DAPI. Original magnification, ×400 (B1–B4). *(* ***C*** *)* Overexpression of HNF4α was detected by immunofluorescence two days after transfection. C1: Green light could be detected from the nuclei of the transfected HNF4α group; C2: Control group transfected with PCDNA3.1. Original magnification, ×400 (C1, C2) *(* ***D*** *)* Overexpression of HNF4α was detected by RT-PCR two days after transfection.

Under hepatic induction conditions, the fibroblastic morphology of HuMSCs gradually changed toward the polygonal shape of hepatocytes with the appearance of abundant granules in the cytoplasm (Fig. 2A).In addition to the morphological differences, we detected hepatocyte-specific marker expression by immunofluorescence. After the HuMSCs were incubated in hepatocyte differentiation medium for 21 days, they became positive for ALB and AFP. The cells cultured in growth medium, which were used as negative controls, were not positively stained for ALB and exhibited low AFP expression (Fig. 2B).

### Transduction of HuMSC-derived hepatoblasts with HNF4α efficiently promotes hepatic maturation

**Figure 3:**
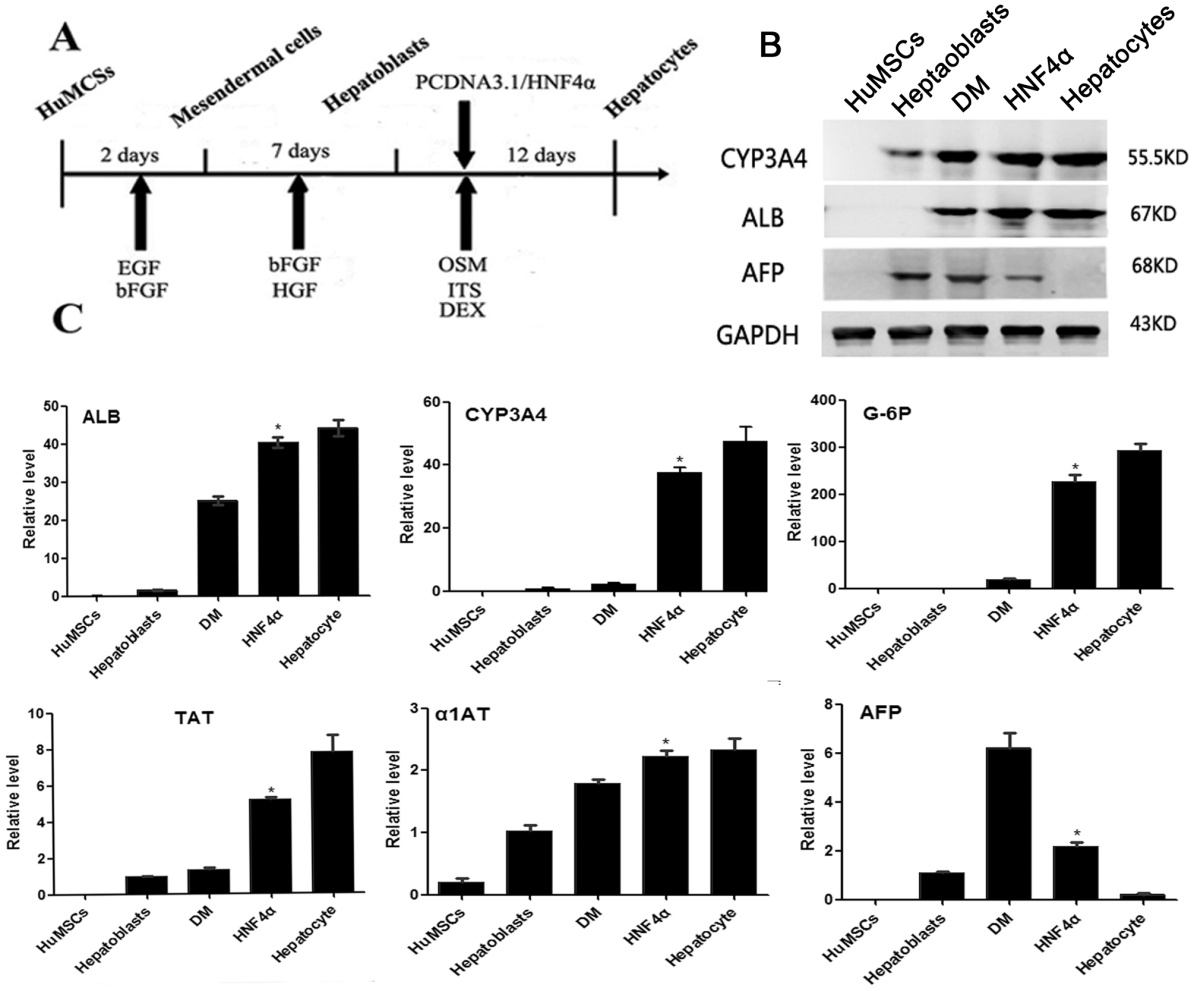
Transfection of hepatoblasts with HNF4α promotes hepatic differentiation. *(* ***A*** *)* The cells, which were cultured for nine days according to the protocol described herein, were transfected with the PCDNA3.1/HNF4α plasmid and cultured for 21 days. *(* ***B*** *)* The protein levels of CYP3A4, ALB and AFP were examined by western blot analysis. *(* ***C*** *)* The gene expression levels of TAT, ALB, CYP3A4, G-6P, α1-antitrypsin and AFP were examined by real-time RT-PCR. All of the data are presented as the means ± SD (n = 3), and the fold induction for each gene induced by HNF4α overexpression was significant (P<0.05). HuMSCs, human umbilical cord mesenchymal stem cells; Hepatoblasts, cells cultured in differentiation medium for nine days; DM, cells cultured in differentiation medium for 21 days and transfected with PCDNA3.1 alone as a control.

**Figure 4:**
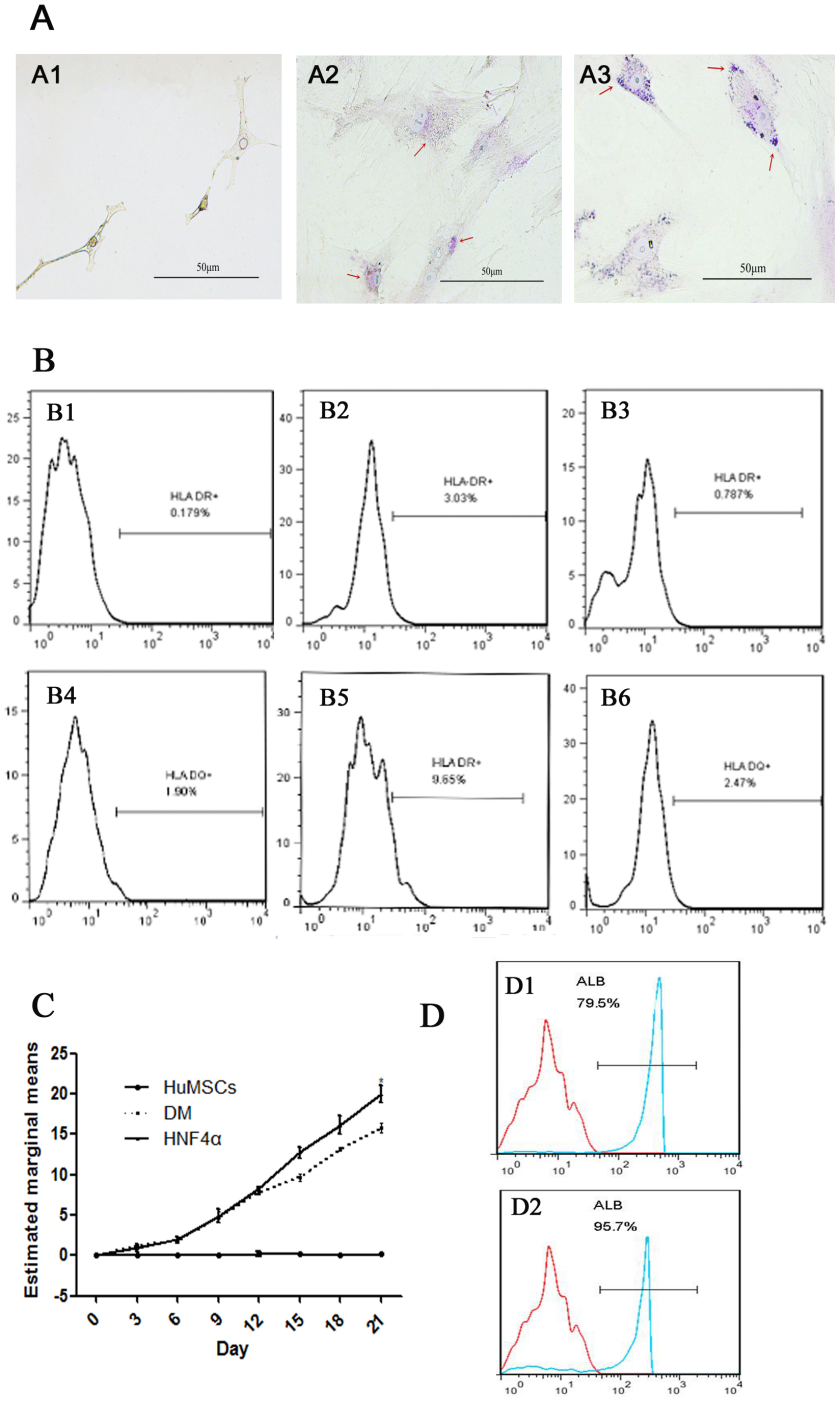
Hallmark function assays of mature hepatocytes. *(* ***A*** *)* Periodic Acid-Schiff staining assay in hepatogenic differentiated cells. A1:HuMSCs; A2: HuMSCs were cultured in hepatocyte differentiation medium for 21 days; A3: HuMSCs were transfected with HNF4α plasmid and cultured until day 21. *(* ***B*** *)* Expression of MHC (HLA-DR and HLA-DQ) assay by flow cytometry analysis. B1,B4:HuMSCs;B2,B5: HuMSCs were cultured in hepatocyte differentiation medium for 21 days transfected with PCDNA3.1 alone;B3,B6: HuMSCs were transfected with HNF4α plasmid and cultured until day 21. *(* ***C, D*** *)* Albumin levels in hepatogenic transfected with HNF4α differentiated cells in comparison to cells cultured in differentiation medium for 21 days transfected with PCDNA3.1 alone by enzyme linked immunosorbent assay (P<0.05) and Flow cytometry analysis (D1: control group D2:HNF4α group).

We aimed to determine whether hepatic maturation is promoted by PCDNA3.1/HNF4α transduction. The human HNF4α gene was introduced into nine-day-induced HuMSCs by plasmid transfection. The overexpression of HNF4α was examined by RT-PCR (Fig. 2D) and immunofluorescence (Fig. 2C) compared to mock control cells infected with PCDNA3.1. The transduced cells were cultured until day 21 of differentiation according to the schematic protocol described in Figure 3A. The control group was transfected with PCDNA3.1 alone and maintained in differentiation medium (DM). We examined the hepatic gene expression on day 21 of differentiation by real-time RT-PCR. The gene expression analysis of TAT, ALB, G-6P, CYP3A4 and α1AT in the transfected HNF4α group revealed higher levels of these genes compared to those found in the cells maintained in hepatic differentiation medium, whereas AFP exhibited lower expression (Fig. 3C). We also examined the protein levels of ALB, AFP and CYP3A4 by western blotting (Fig. 3B), and the results were consistent with the real-time RT-PCR findings. Upon treatment with PCDNA3.1/HNF4α transduction, glycogen uptake was detected after 21 days by Periodic Acid-Schiff (PAS) staining (Fig. 4A). Intracellular glycogen in the transfected HNF4α group (Fig. 4A3) was higher levels compared to control group (Fig. 4A2). Both groups were negative for MHC (HLA-DR and HLA-DQ) after 21days (Fig. 4B). Albumin was secreted in the culture media and was analyzed on days 0, 3, 6, 9, 12, 15, 18 and 21 of differentiation. We examined the transfected HNF4α group produced significantly higher levels of albumin compared to control group from about 12 days of differentiation (Fig. 4C). From the results of FACS, about 98% of the cells in transfected HNF4α group expressing ALB, whereas the control group was 82% (Fig. 4D).

### HNF4α promotes hepatic differentiation by regulating liver-enriched factors

**Figure 5:**
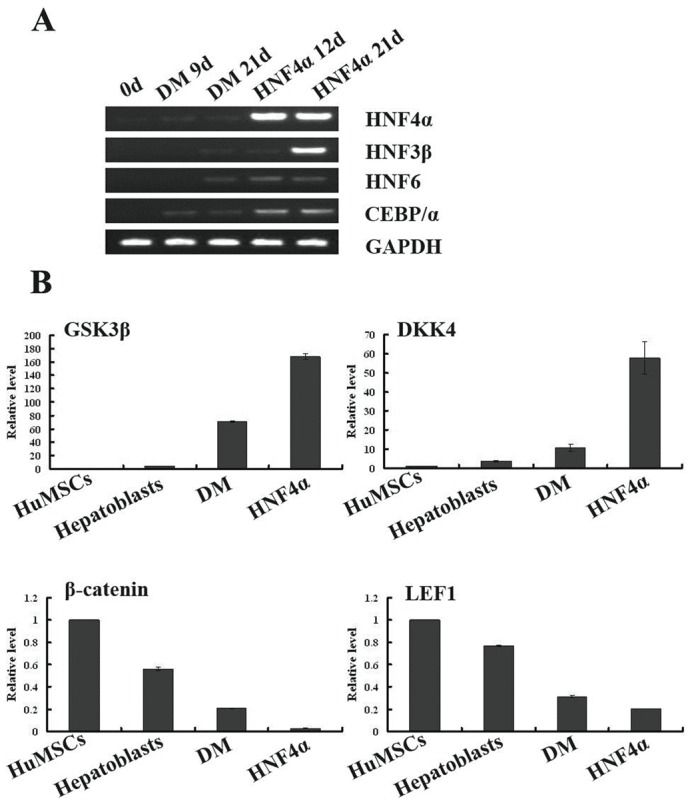
Mechanism of HNF4α-mediated improved hepatic differentiation. *(* ***A*** *)* Detection of liver-enriched transcription factors in induced and HNF4α-transfected HuMSCs by RT-PCR. 0 d: HuMSCs before induction; DM 9 d and 21 d: Cells cultured in hepatic differentiation medium for nine days and 21 days, respectively; HNF4α 12 d and 21 d: Cells transfected with HNF4α nine days after induction (gene expression was determined three and 12 days later, respectively). *(* ***B*** *)* Wnt/β-catenin signaling-related genes were examined by real-time RT-PCR. All of the data are presented as the means ± SD (n = 3), and the fold induction for each gene by HNF4α overexpression was significant (P<0.05).

Several studies have demonstrated that hepatic gene expression is regulated by the combinational action of liver-enriched factors. In this study, we observed weak expression of HNF4α, HNF3β, HNF6 and CEBP/α in the cells cultured in differentiation medium. HNF4α is crucial for the expression of hepatic genes, including liver-enriched transcription factors [9]. Therefore, we investigated whether the transcriptional efficiency of liver-enriched factors was regulated by HNF4α overexpression in the induced HuMSCs. The expression levels of additional liver-enriched transcription factors in the HNF4α-transfected cells were examined by RT-PCR analysis. HNF6 and CEBP/α exhibited robust expression three days after transfection. This increase continued for 12 days after transfection, and HNF3β began to be expressed at high levels (Fig. 5A).Besides, we also analysis of Wnt/β-catenin in hepatocyte-like cells after HNF4α transfection. Because the Wnt/β-**c**atenin pathway plays a fundamental role in the control of adult stem cell differentiation, we analyzed the expression of several genes regulated by this pathway, such as β-**c**atenin, glycogen synthase kinase 3 beta (GSK3β), dickkopf 4 (DKK4) and frizzled 2 (FZD2). The results indicated that this pathway was suppressed upon induction in hepatic differentiation medium, and this effect was more pronounced after HNF4α transfection (Fig. 5B).

## Discussion

Many reports have demonstrated the pluripotency and low immunogenicity of HuMSCs; thus, these cells are expected to be a source of specific cell types for transplantation. The phenotypic profile of the HuMSCs isolated in our study was consistent with that found in previous studies and the documented expression of the consensus MSC marker set. Indeed, the HuMSCs were positive for CD90, CD105, CD13 and CD59 and negative for CD45, CD34 and HLA-DR (Fig. 1B). The HuMSCs were examined for their ability to undergo adipogenic and pancreatic differentiation to illustrate their multipotent differentiation potential for not only mesodermal but also endodermal differentiation. Our results also demonstrated that the HuMSCs were well differentiated (Fig. 1A).

The hepatic differentiation of HuMSCs has been reported by the addition of various growth factors and cytokines. In this study, we induced the hepatic differentiation of HuMSCs according to the protocol described by Lee et al. [14]. The hepatic gene expression pattern in the induced MSCs appears to be correlated with the developmental process of the liver in vivo. We found that, upon exposure to the differentiation medium for two weeks, the cells began to form clusters (Fig. 2A). Then, the HuMSCs gradually progressed toward the polygonal morphology of mature hepatocytes and expressed liver-specific protein markers, such as ALB and AFP (Fig. 2B). However, the differentiation efficiency remains insufficient for clinical application. Therefore, we attempted to achieve transdifferentiation with high efficiency by overexpressing HNF4α, which plays an important part in hepatic differentiation. HNF4α is initially expressed in the developing hepatic diverticulum on E8.75 [18], [19], and its expression is elevated as the liver develops. A previous loss-of-function study showed that HNF4α plays a critical role in liver development; the conditional deletion of HNF4α in fetal hepatocytes results in the faint expression of many mature enzymes and the impairment of normal liver morphology [8]. Our study first focused on the function of HNF4α in the hepatic differentiation of HuMSCs.

According to Takayama et al. [13], we chose the optimal time for HNF4α transfection as the time at which the cells were induced into hepatoblasts. Because endogenous HNF4α is initially expressed in the hepatoblasts [18], [19], our system might adequately reflect early embryogenesis (Fig. 3A). We found that the expression levels of the functional hepatic genes TAT, ALB, CYP3A4, G-6P and α1-antitrypsin were upregulated by HNF4α transfection compared to cells differentiated only in hepatic differentiation medium, whereas AFP exhibited lower expression, indicating a higher degree of hepatocyte maturation (Fig. 3C). The results of the western blot analyses of the ALB, CYP3A4 and AFP proteins were consistent with the real-time RT-PCR findings.

It is well known that several liver-enriched transcription factors can coordinately regulate the expression of hepatic genes involved in liver-specific functions [20], [21]. Among them, HNF4α may act as a master gene in the transcriptional cascade that regulates the constitutive expression of target genes. Therefore, we investigated the transcriptional efficiency of liver-enriched factors regulated by HNF4α overexpression in induced HuMSCs. Interestingly, we found that the HuMSCs weakly expressed HNF4α but did not express other liver-enriched factors.

Previous studies have demonstrated that HNF4α is expressed not only in the liver but also in the kidney, heart, spleen and intestine, although at different levels [22], [23]. The overexpression of HNF4α before hepatic specification may promote bidirectional differentiation and a heterogeneous population. Therefore, we chose the appropriate time at which HNF4α could participate in the liver-enriched factor network. After the cells were induced in hepatic differentiation medium for nine days, the liver-enriched factors gradually began to be expressed (Fig. 4A). However, the factors were still weakly expressed until 21 days after induction. The overexpression of HNF4α upregulated the expression of HNF6, CEBP/α and HNF3β after transfection, as determined by the RT-PCR analysis results. Therefore, we concluded that HNF4α might promote hepatic differentiation by regulating liver-enriched factors. Wnt/β-catenin is activated as mesenchymal stem cells differentiate into osteoblasts [24] and is inactivated during differentiation into adipocytes [25]. Previous reports have shown that the down-regulation of Wnt/β-catenin signaling plays a role in the hepatic differentiation of MSCs [26]–[28]. We also focused on Wnt/β-catenin signals as one of the important mechanisms for hepatic differentiation induced by HNF4α overexpression. Our results showed that the overexpression of HNF4α suppressed this pathway to a greater extent than observed in cells induced in hepatic differentiation medium alone. Thus, the improved hepatic differentiation of HuMSCs caused by HNF4α may be associated with the inactivation of Wnt/β-catenin signals.

In summary, our data demonstrate that HuMSCs can be easily induced into hepatocyte-like cells under hepatic differentiation conditions. Furthermore, HNF4α is a key factor in determining the differentiation status of the hepatocyte-like cells derived from HuMSCs. The overexpression of HNF4α can activate various hepatic-specific genes and enhance the differentiation status of differentiated HuMSCs. This research provides an experimental basis for further research in stem cell transplantation, and stem cell transplantation will provide a broad scope in future clinical applications for liver regeneration after hepatectomy and living donor liver transplantation (LDLT). This investigation may also provide the means to generate reliable cell sources for bioartificial liver support devices and hepatocyte transplantation in the future. However, several limitations, including genomic integration into target cells and the cytotoxicity of target cells, have impeded the clinical utility of these methods. Therefore, solutions should be considered in future studies.
